# Supplementary figures and images for: Prevalent and dynamic binding of the cell cycle checkpoint kinase Rad53 to gene promoters
Source: eLife. 2022 Dec 15;11:e84320. doi: 10.7554/eLife.84320 (PMC9797190; doi:10.7554/eLife.84320)

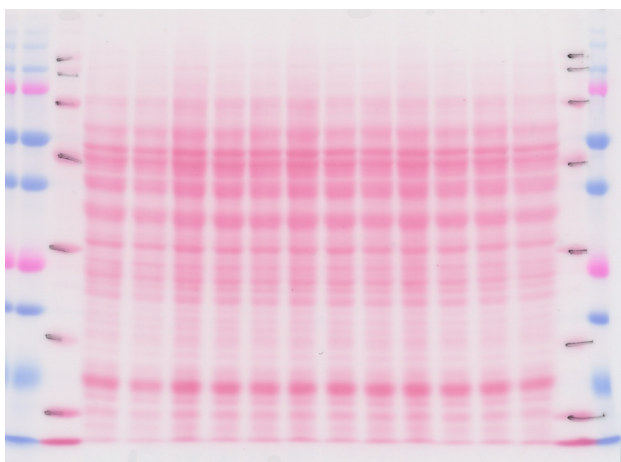

Supplement: Figure 2—figure supplement 1—source data 10. [file elife-84320-fig2-figsupp1-data10.zip › Figure 2 supplement 1A 84320 /Figure 2 supplement 1a-source data 1486.pdf]

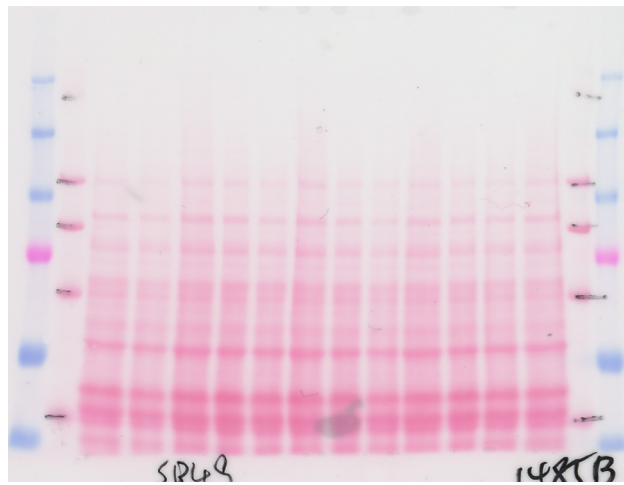

Supplement: Figure 2—figure supplement 1—source data 10. [file elife-84320-fig2-figsupp1-data10.zip › Figure 2 supplement 1A 84320 /Figure 2 supplement 1a-source data 1485.pdf]

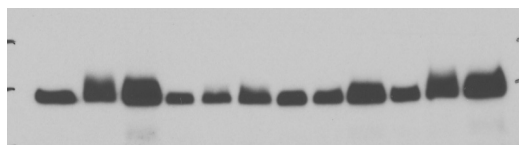

Supplement: Figure 2—figure supplement 1—source data 10. [file elife-84320-fig2-figsupp1-data10.zip › Figure 2 supplement 1A 84320 /Figure 2 supplement 1a-source data 1485A wb1 104232.pdf]

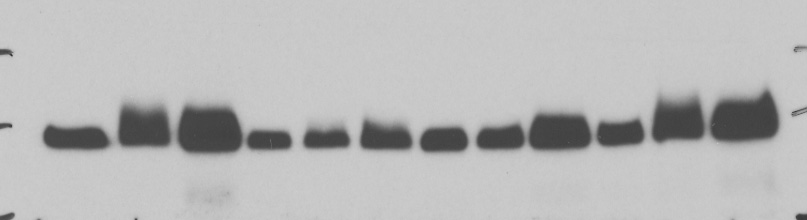

Supplement: Figure 2—figure supplement 1—source data 10. [file elife-84320-fig2-figsupp1-data10.zip › Figure 2 supplement 1A 84320 /1485A wb1 104232.jpg]

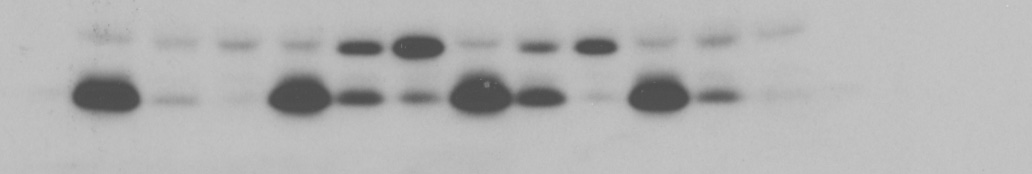

Supplement: Figure 2—figure supplement 1—source data 10. [file elife-84320-fig2-figsupp1-data10.zip › Figure 2 supplement 1A 84320 /1486 wb2 sml1.jpg]

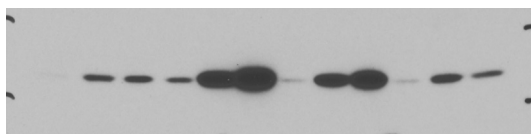

Supplement: Figure 2—figure supplement 1—source data 10. [file elife-84320-fig2-figsupp1-data10.zip › Figure 2 supplement 1A 84320 /Figure 2 supplement 1a-source data 1486 wb1 S129.pdf]

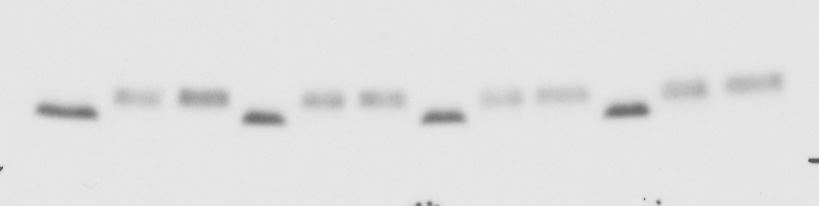

Supplement: Figure 2—figure supplement 1—source data 10. [file elife-84320-fig2-figsupp1-data10.zip › Figure 2 supplement 1A 84320 /1485B wb1 SB49.jpg]

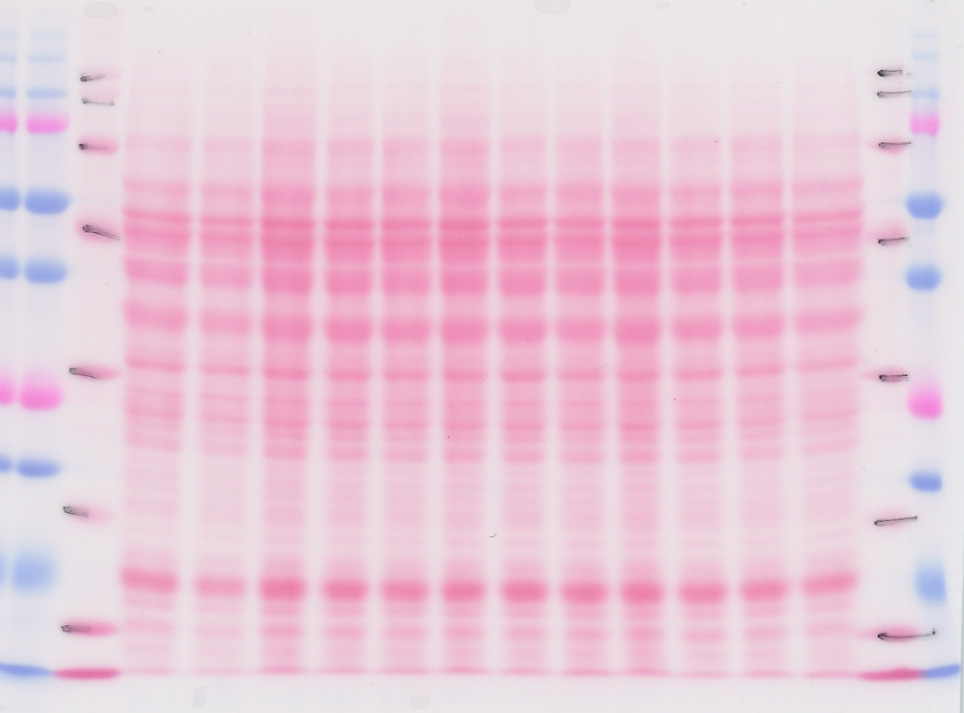

Supplement: Figure 2—figure supplement 1—source data 10. [file elife-84320-fig2-figsupp1-data10.zip › Figure 2 supplement 1A 84320 /1486.jpg]

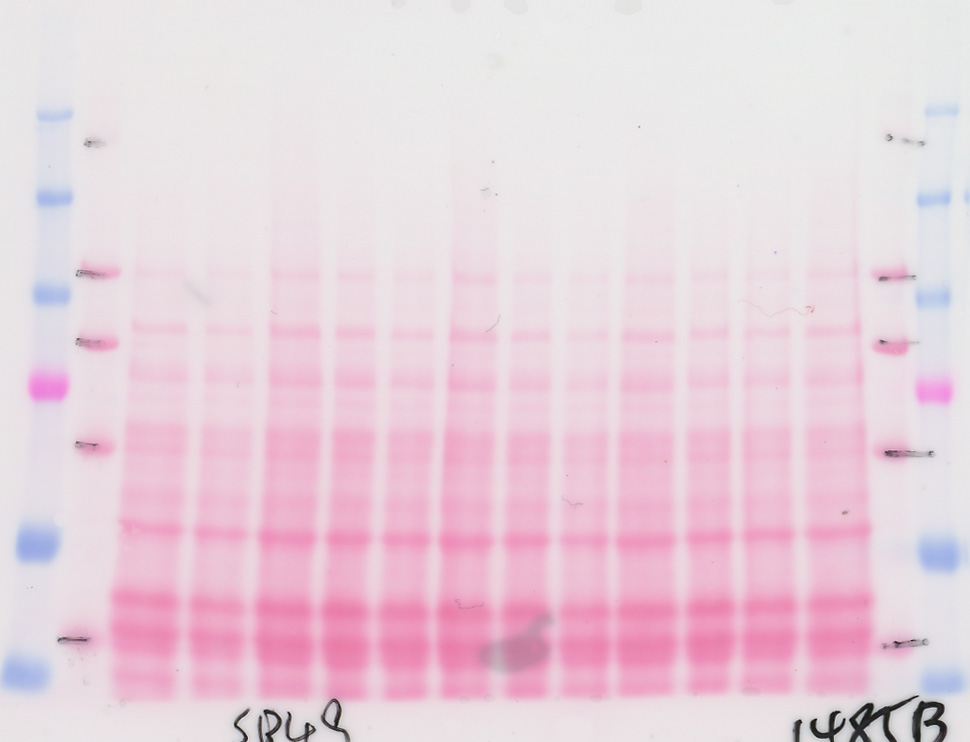

Supplement: Figure 2—figure supplement 1—source data 10. [file elife-84320-fig2-figsupp1-data10.zip › Figure 2 supplement 1A 84320 /1485.jpg]

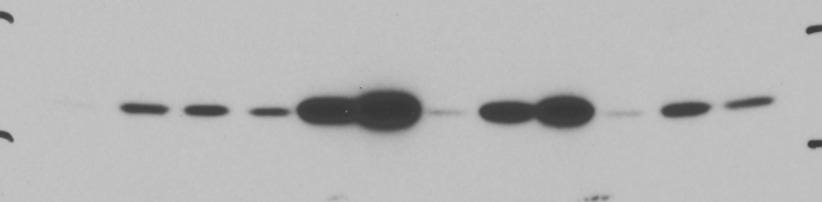

Supplement: Figure 2—figure supplement 1—source data 10. [file elife-84320-fig2-figsupp1-data10.zip › Figure 2 supplement 1A 84320 /1486 wb1 S129.jpg]

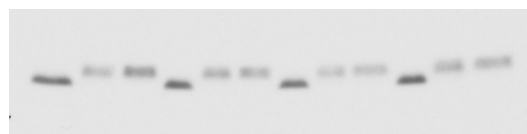

Supplement: Figure 2—figure supplement 1—source data 10. [file elife-84320-fig2-figsupp1-data10.zip › Figure 2 supplement 1A 84320 /Figure 2 supplement 1a-source data 1485B wb1 SB49.pdf]

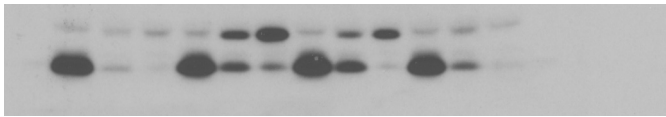

Supplement: Figure 2—figure supplement 1—source data 10. [file elife-84320-fig2-figsupp1-data10.zip › Figure 2 supplement 1A 84320 /Figure 2 supplement 1a-source data 1486 wb2 sml1.pdf]

Source Figure 2 – figure supplement 1a

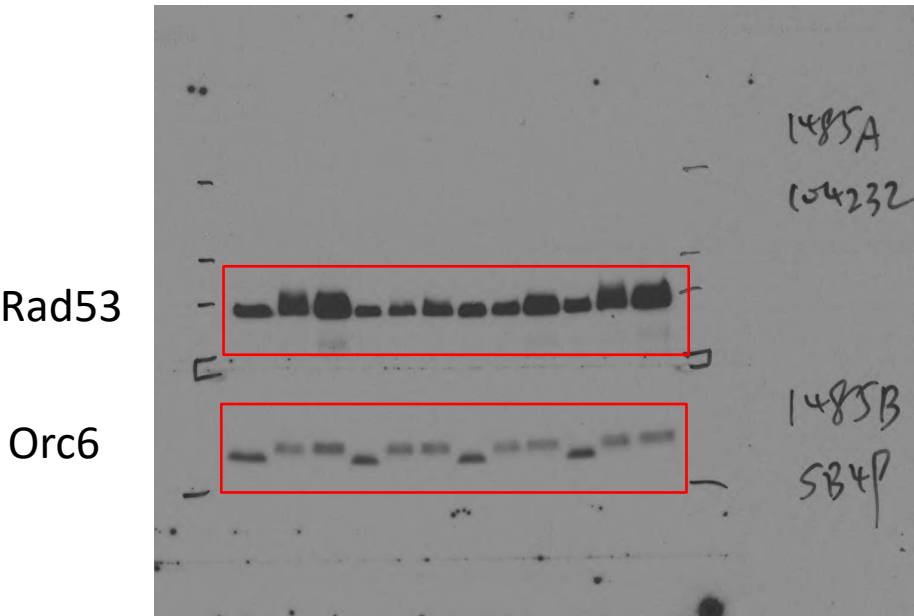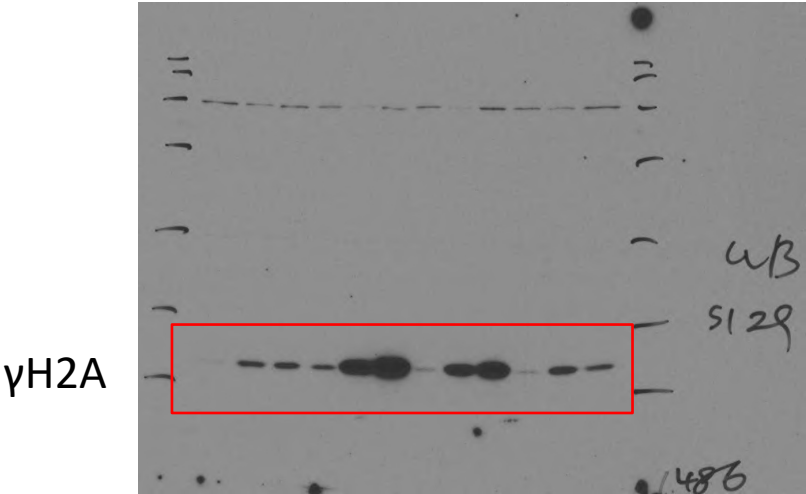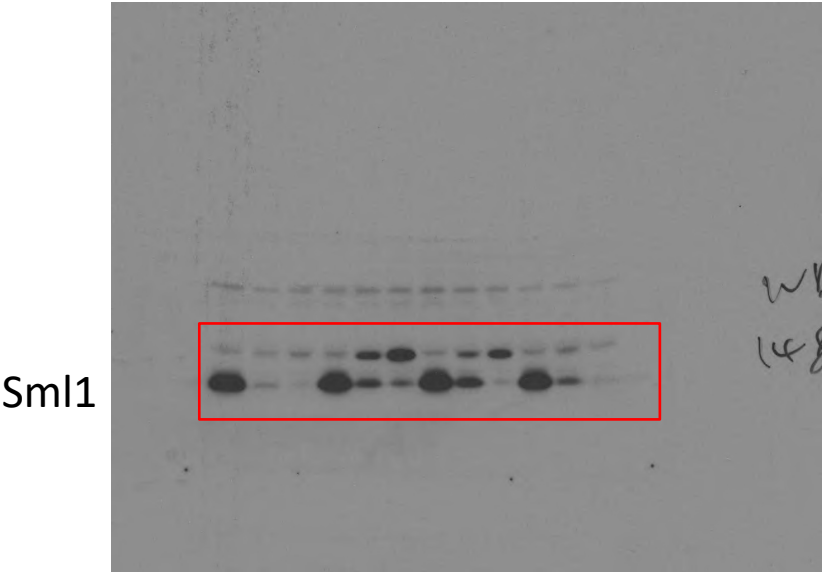

Supplement: Figure 2—figure supplement 1—source data 10. [file elife-84320-fig2-figsupp1-data10.zip › Figure 2 supplement 1A 84320 /Source for Figure 2 ΓÇô figure supplement 1a.pdf]

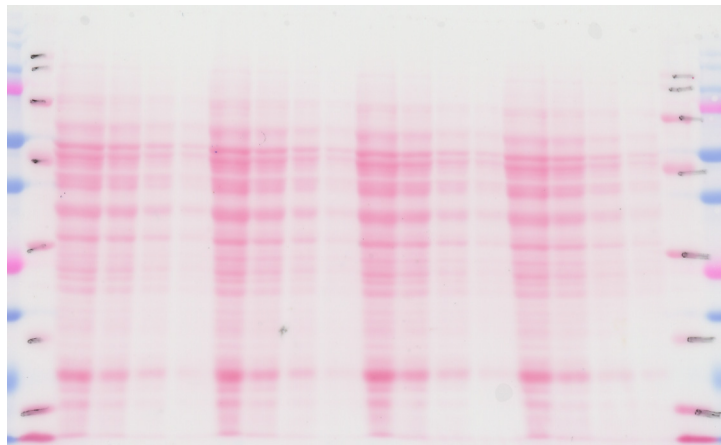

Supplement: Figure 2—figure supplement 1—source data 11. [file elife-84320-fig2-figsupp1-data11.zip › Figure 2 supplement 1B 84320/Figure 2 supplement 1 b-source data 1490.pdf]

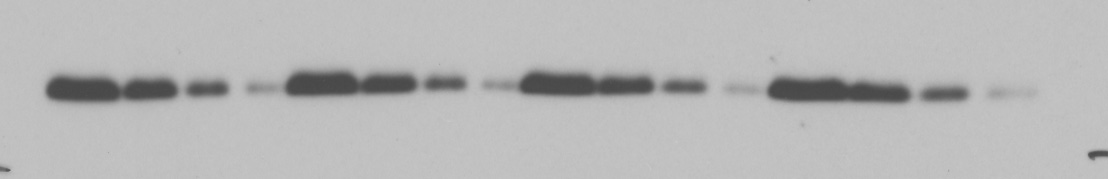

Supplement: Figure 2—figure supplement 1—source data 11. [file elife-84320-fig2-figsupp1-data11.zip › Figure 2 supplement 1B 84320/1489B wb1 SB49 M.jpg]

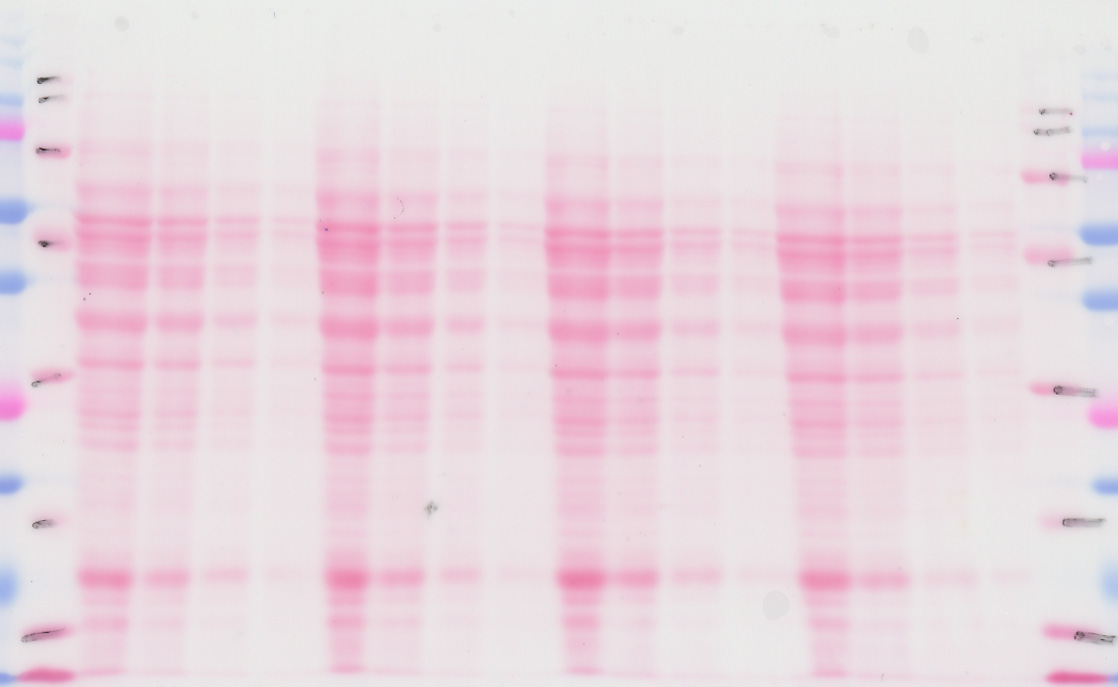

Supplement: Figure 2—figure supplement 1—source data 11. [file elife-84320-fig2-figsupp1-data11.zip › Figure 2 supplement 1B 84320/1490.jpg]

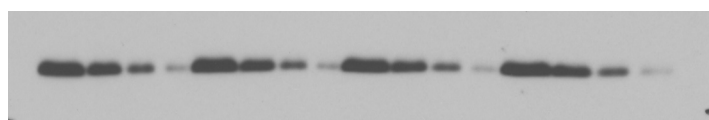

Supplement: Figure 2—figure supplement 1—source data 11. [file elife-84320-fig2-figsupp1-data11.zip › Figure 2 supplement 1B 84320/Figure 2 supplement 1 b-source data 1489B wb1 SB49 M.pdf]

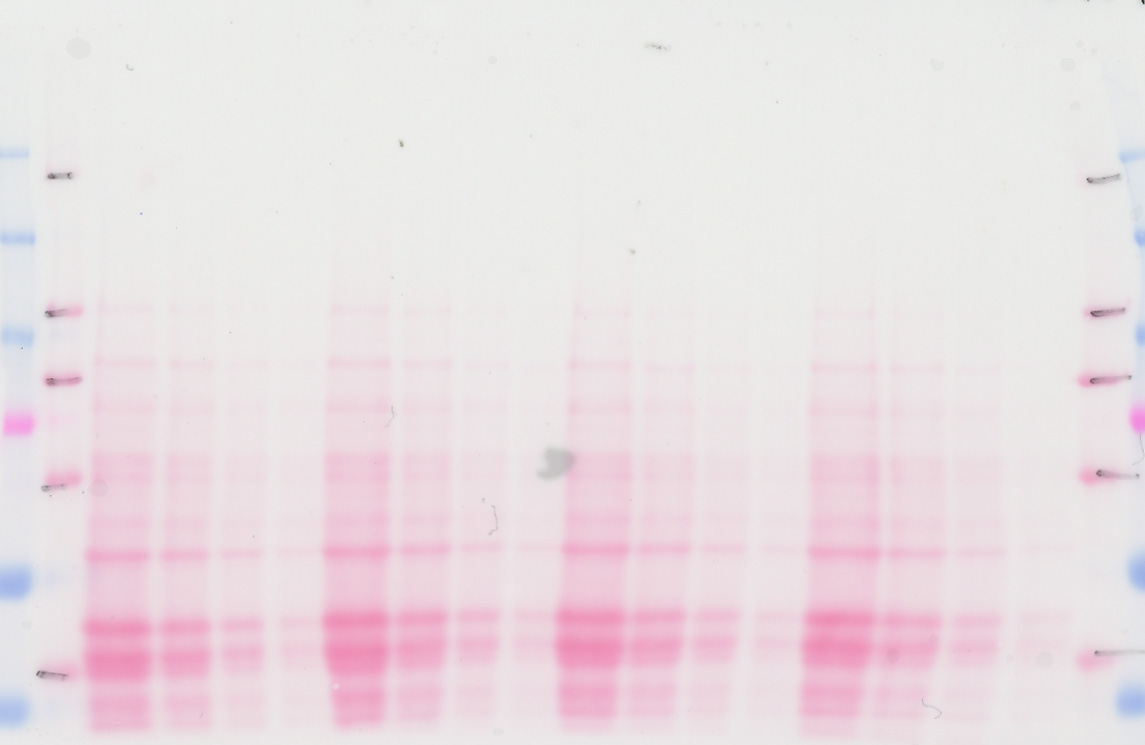

Supplement: Figure 2—figure supplement 1—source data 11. [file elife-84320-fig2-figsupp1-data11.zip › Figure 2 supplement 1B 84320/1489.jpg]

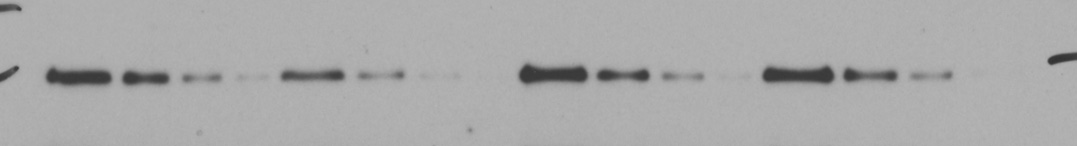

Supplement: Figure 2—figure supplement 1—source data 11. [file elife-84320-fig2-figsupp1-data11.zip › Figure 2 supplement 1B 84320/1489A wb1 m166859.jpg]

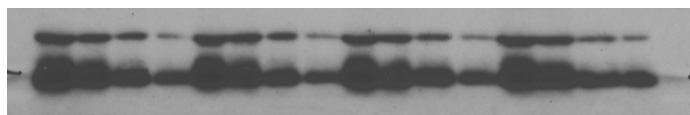

Supplement: Figure 2—figure supplement 1—source data 11. [file elife-84320-fig2-figsupp1-data11.zip › Figure 2 supplement 1B 84320/Figure 2 supplement 1 b-source data 1490B wb2 sml1.pdf]

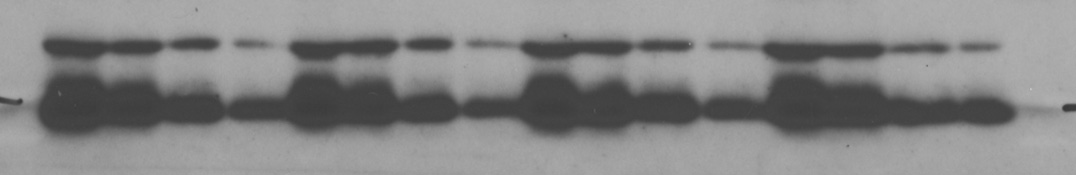

Supplement: Figure 2—figure supplement 1—source data 11. [file elife-84320-fig2-figsupp1-data11.zip › Figure 2 supplement 1B 84320/1490B wb2 sml1.jpg]

Source Figure 2 – figure supplement 1b

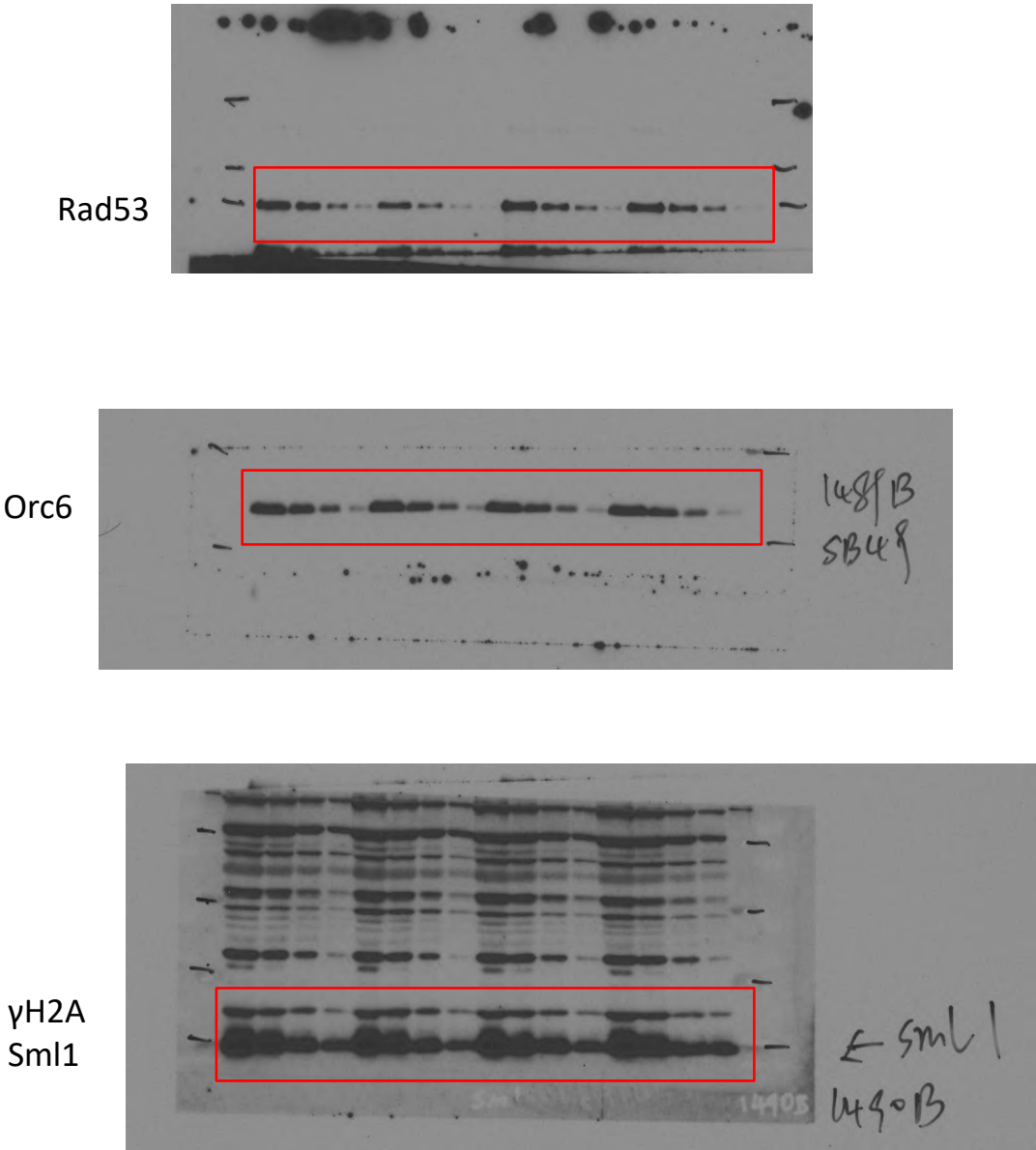

Supplement: Figure 2—figure supplement 1—source data 11. [file elife-84320-fig2-figsupp1-data11.zip › Figure 2 supplement 1B 84320/Source for Figure 2 ΓÇô figure supplement 1b.pdf]

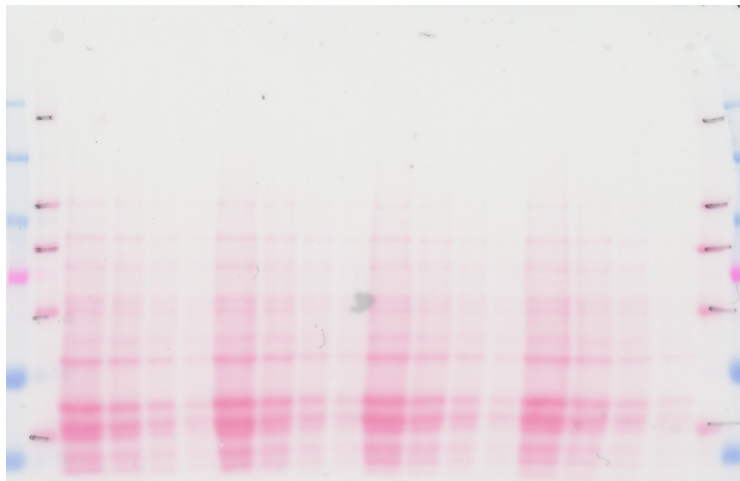

Supplement: Figure 2—figure supplement 1—source data 11. [file elife-84320-fig2-figsupp1-data11.zip › Figure 2 supplement 1B 84320/Figure 2 supplement 1 b-source data 1489.pdf]

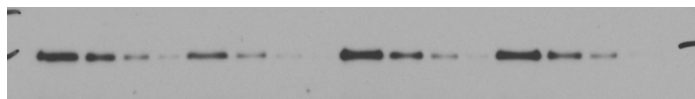

Supplement: Figure 2—figure supplement 1—source data 11. [file elife-84320-fig2-figsupp1-data11.zip › Figure 2 supplement 1B 84320/Figure 2 supplement 1 b-source data 1489A wb1 m166859.pdf]

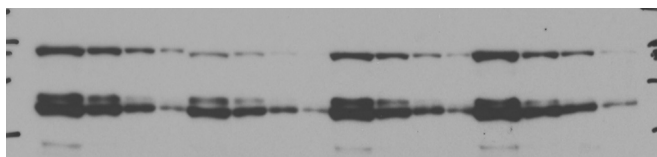

Supplement: Figure 2—figure supplement 1—source data 12. [file elife-84320-fig2-figsupp1-data12.zip › Figure 2 supplement 1C 84320/Figure 2 supplement 1c-source data 1496A wb1 104232.pdf]

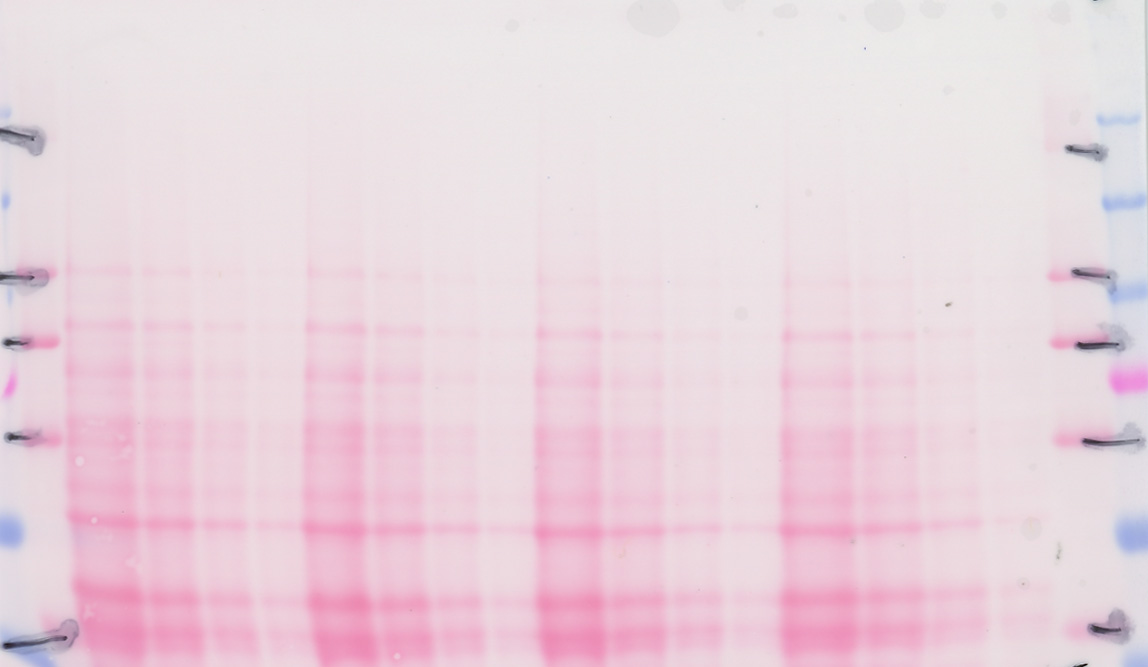

Supplement: Figure 2—figure supplement 1—source data 12. [file elife-84320-fig2-figsupp1-data12.zip › Figure 2 supplement 1C 84320/1495.jpg]

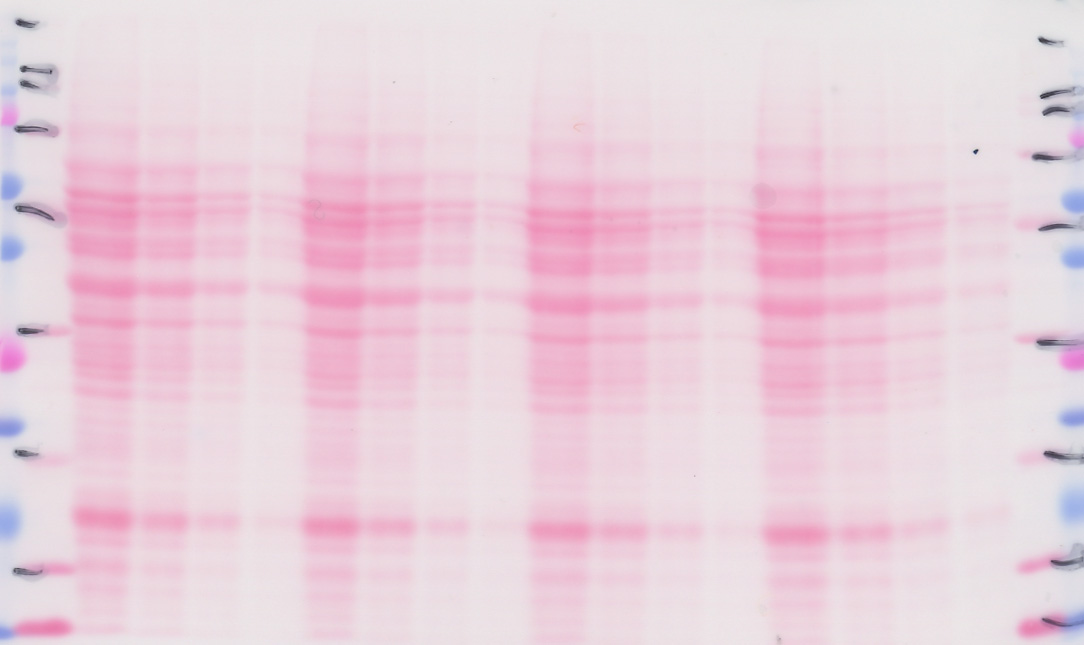

Supplement: Figure 2—figure supplement 1—source data 12. [file elife-84320-fig2-figsupp1-data12.zip › Figure 2 supplement 1C 84320/1496.jpg]

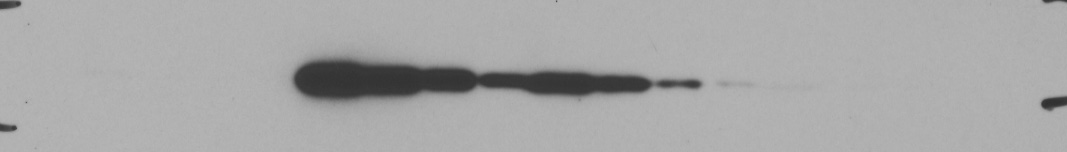

Supplement: Figure 2—figure supplement 1—source data 12. [file elife-84320-fig2-figsupp1-data12.zip › Figure 2 supplement 1C 84320/1496B wb1 S129 L.jpg]

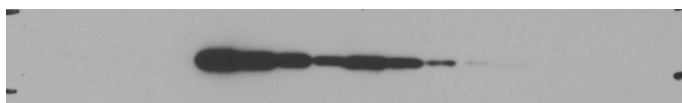

Supplement: Figure 2—figure supplement 1—source data 12. [file elife-84320-fig2-figsupp1-data12.zip › Figure 2 supplement 1C 84320/Figure 2 supplement 1c-source data 1496B wb1 S129 L.pdf]

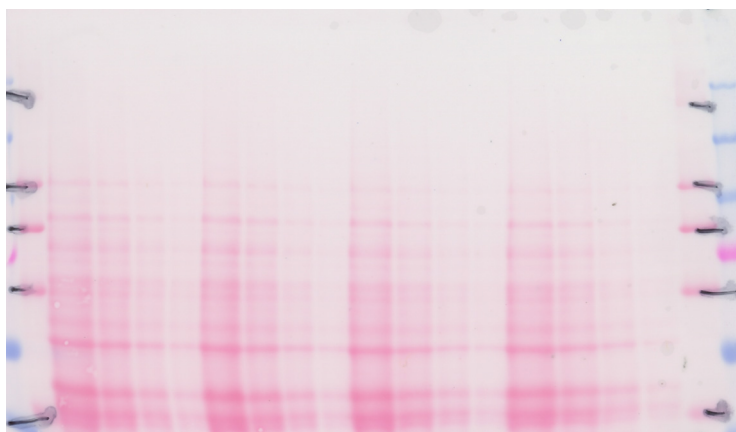

Supplement: Figure 2—figure supplement 1—source data 12. [file elife-84320-fig2-figsupp1-data12.zip › Figure 2 supplement 1C 84320/Figure 2 supplement 1c-source data 1495.pdf]

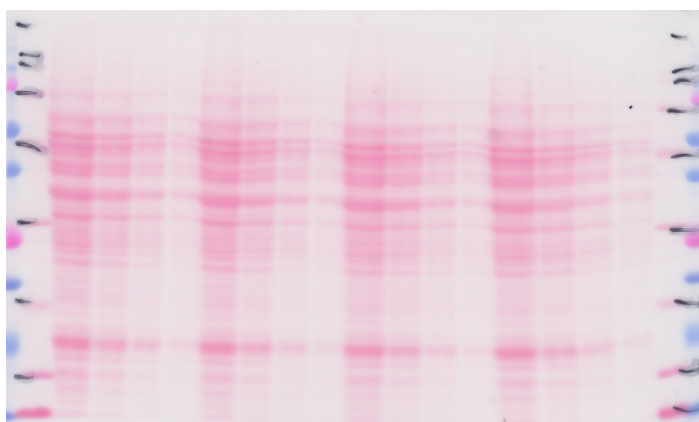

Supplement: Figure 2—figure supplement 1—source data 12. [file elife-84320-fig2-figsupp1-data12.zip › Figure 2 supplement 1C 84320/Figure 2 supplement 1c-source data 1496.pdf]

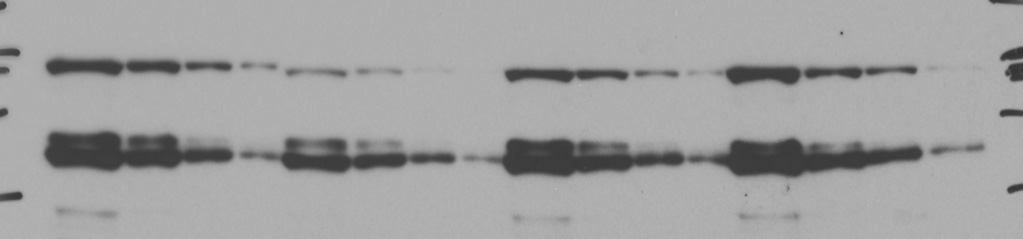

Supplement: Figure 2—figure supplement 1—source data 12. [file elife-84320-fig2-figsupp1-data12.zip › Figure 2 supplement 1C 84320/1496A wb1 104232.jpg]

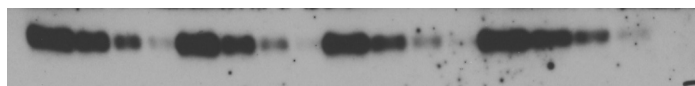

Supplement: Figure 2—figure supplement 1—source data 12. [file elife-84320-fig2-figsupp1-data12.zip › Figure 2 supplement 1C 84320/Figure 2 supplement 1 c-source data 1495B wb1 SB49 M.pdf]

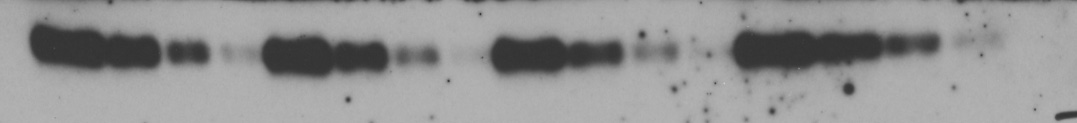

Supplement: Figure 2—figure supplement 1—source data 12. [file elife-84320-fig2-figsupp1-data12.zip › Figure 2 supplement 1C 84320/1495B wb1 SB49 M.jpg]

Source Figure 2 – figure supplement 1c

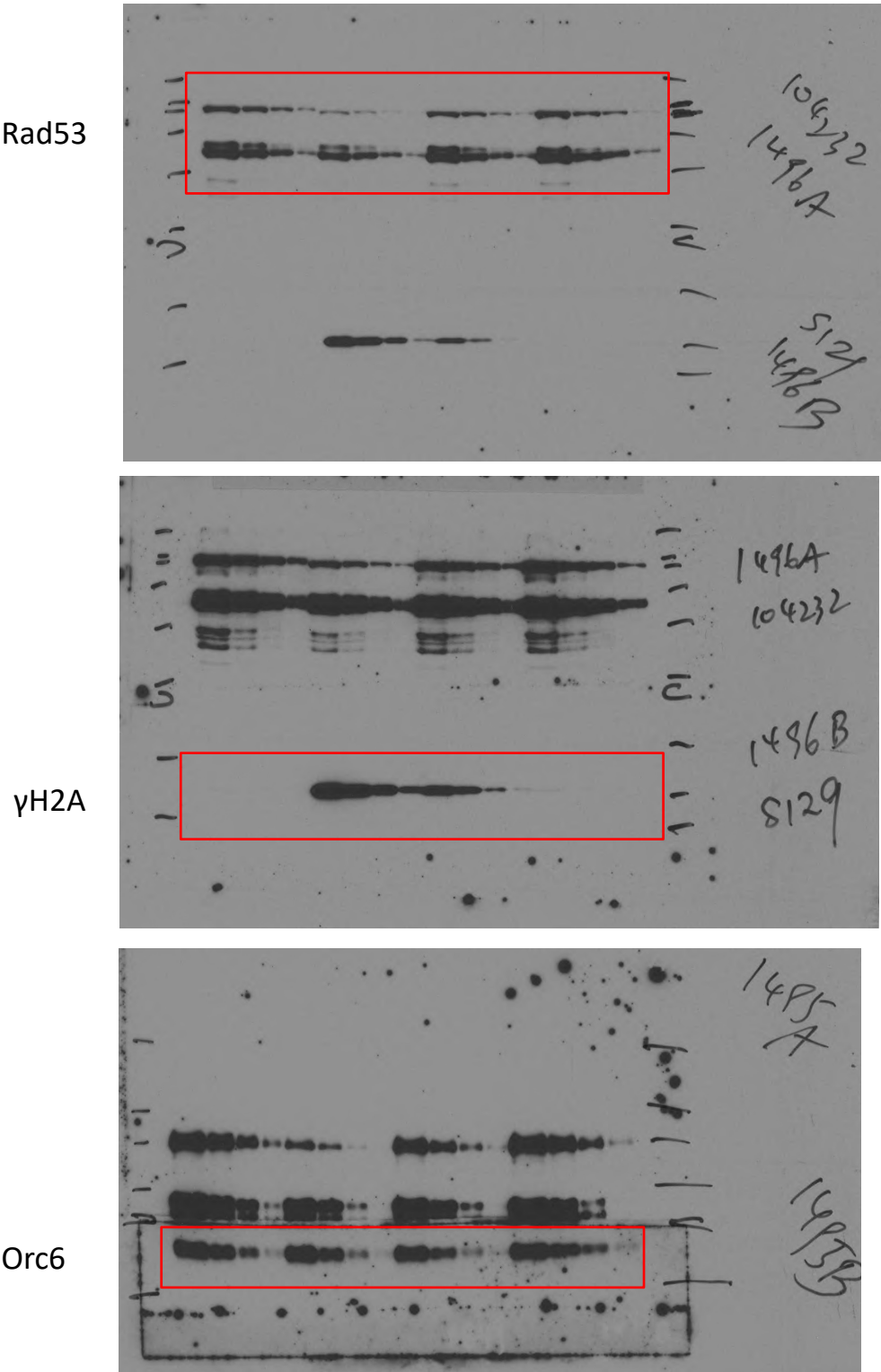

Supplement: Figure 2—figure supplement 1—source data 12. [file elife-84320-fig2-figsupp1-data12.zip › Figure 2 supplement 1C 84320/Source for Figure 2 ΓÇô figure supplement 1c.pdf]
